# Quantitative evaluation of deep convolutional neural network-based image denoising for low-dose computed tomography

**DOI:** 10.1186/s42492-021-00087-9

**Published:** 2021-07-25

**Authors:** Keisuke Usui, Koichi Ogawa, Masami Goto, Yasuaki Sakano, Shinsuke Kyougoku, Hiroyuki Daida

**Affiliations:** 1 Department of Radiological Technology, Faculty of Health Science, Juntendo University, Tokyo, 113-8421 Japan; 2 Department of Radiation Oncology, Faculty of Medicine, Juntendo University, Tokyo, 113-8421 Japan; 3 Faculty of Science and Engineering, Hosei University, Tokyo, 184-8584 Japan

**Keywords:** Deep learning, Convolutional neural network, Low-dose computed tomography, Denoising, Image quality

## Abstract

To minimize radiation risk, dose reduction is important in the diagnostic and therapeutic applications of computed tomography (CT). However, image noise degrades image quality owing to the reduced X-ray dose and a possible unacceptably reduced diagnostic performance. Deep learning approaches with convolutional neural networks (CNNs) have been proposed for natural image denoising; however, these approaches might introduce image blurring or loss of original gradients. The aim of this study was to compare the dose-dependent properties of a CNN-based denoising method for low-dose CT with those of other noise-reduction methods on unique CT noise-simulation images. To simulate a low-dose CT image, a Poisson noise distribution was introduced to normal-dose images while convoluting the CT unit-specific modulation transfer function. An abdominal CT of 100 images obtained from a public database was adopted, and simulated dose-reduction images were created from the original dose at equal 10-step dose-reduction intervals with a final dose of 1/100. These images were denoised using the denoising network structure of CNN (DnCNN) as the general CNN model and for transfer learning. To evaluate the image quality, image similarities determined by the structural similarity index (SSIM) and peak signal-to-noise ratio (PSNR) were calculated for the denoised images. Significantly better denoising, in terms of SSIM and PSNR, was achieved by the DnCNN than by other image denoising methods, especially at the ultra-low-dose levels used to generate the 10% and 5% dose-equivalent images. Moreover, the developed CNN model can eliminate noise and maintain image sharpness at these dose levels and improve SSIM by approximately 10% from that of the original method. In contrast, under small dose-reduction conditions, this model also led to excessive smoothing of the images. In quantitative evaluations, the CNN denoising method improved the low-dose CT and prevented over-smoothing by tailoring the CNN model.

## Introduction

Computed tomography (CT) is widely used for repetitive screening diagnostic scans, such as scans for cancer, lung nodules, and bleeding internal organs. High-exposure scans can cause patients to suffer from several biological effects that increase the risk of cancer [1, 2]. To minimize exposure doses, reducing the number of X-ray photons via tube-current modulation is a viable solution. However, this method is limited because low-dose CT triggers image-quality degradation. Hence, lower doses of X-ray photons provide noisier reconstructed CT images, which may reduce the diagnostic performance to an unacceptable level [3].

To address this image degradation, several techniques have been proposed to improve the quality of low-dose CT images. Sinogram domain filtration and iterative reconstruction methods combine the statistical properties of the data in the image domain and projection space to optimize the objective function. Although these methods can eliminate image noise, they depend on the specifications of the manufacturer, thereby limiting their clinical applications. Computational costs are also required; hence, the reconstruction process is relatively slow [4, 5]. Moreover, image-space denoising methods, such as median, Gaussian, and Wiener filters, do not require projection data and aim to reduce image noise without requiring an understanding of the structures of interest. Therefore, they are exposed to the risk of either generating new image artifacts or losing original structural information during post-processing, which limit their clinical applications [6].

Deep learning approaches have been widely used for image denoising, and convolutional neural networks (CNNs), which are based on extensive data and powerful graphics-processing units, have achieved substantial success [7, 8]. Several CNN-based methods have been proposed for natural image denoising and low-dose CT. However, these CNNs are often inhibited by disappearing gradients and the introduction of image blurring, which leads to difficulties in training [9]. A denoising network (Dn), known as DnCNN, incorporates residual learning and batch normalization (BN), and can yield better improvements for Gaussian denoising tasks with unknown noise. In this technique, residual learning can be realized by improving the training efficiency when the input and output are close to each other, such as a noise image [10]. Moreover, BN facilitates the application of significantly higher learning rates by stabilizing the training process, which can address the vanishing gradient challenge in these deeper learning processes [11].

However, this method has been demonstrated to be inferior to more realistic and complicated noises [10, 12]. Several CNN-based methods have been evaluated for clinical low-dose CT denoising. Chen et al. [13] demonstrated the potential of a CNN-based framework for artifact reduction and structure preservation in low-dose CT imaging. Deep learning algorithms that adopt residual encoder-decoder networks have been evaluated and validated for CT denoising, and these networks have exhibited significant potential for noise suppression, structural preservation, and lesion detection at a high computational speed [14]. However, the dependence of the incident dose reduction in the CNN-based denoising method has not been elucidated, and dose reduction levels for the effective operation of the CNN model need to be validated.

In this study, to better understand the dose-dependent properties of the CNN-based method, DnCNN was implemented to denoise low-dose CT images, and compared the image quality with that of other noise-reduction methods on unique CT noise simulation images. Collecting real human images obtained at various incident dose levels to train a deep learning model significantly increases the cost of the learning process. To address this challenge, the aim of this study was to simulate virtual low-dose images at several dose-reduction levels, and apply physical evaluation metrics of image quality to quantitatively compare the noise-reduction performance of different denoising methods on simulated images.

## Methods

### Network architecture

**Fig. 1:**
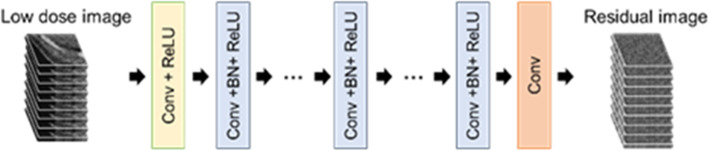
Overall architecture of the DnCNN with simulated-noise images. This network learns residual mapping for noise image. The main modules include convolutional filtering, ReLU and BN

The network structure of the DnCNN was adopted as the general CNN model [10]. This model was pretrained on 400 Gy-level images with a Gaussian noise level *σ* = 25 for natural image denoising. The input image size for training was 50 × 50 pixels, the convolution layers were set to 20, and 64 convolutional filters were used at a size of 3 × 3 pixels to generate the feature maps. Residual learning and BN were used to enhance denoising performance and learning speed. Moreover, defined as the positive part of its value, rectified linear units (ReLU) were adopted for faster training of deep neural networks. DnCNN learns residual map data and generates a noise-reduced image, which can yield more efficient training and accurate results within very deep networks. BN was applied between the convolution filters, and ReLU was used in all middle layers to enable higher learning rates with the normalization of each sub-sample set. The architecture of the DnCNN network is illustrated in Fig. 1.

### Transfer learning

The concept of the proposed transfer learning is to update the pretrained model of the DnCNN for adapting CT-specific dose-reduction images. In this study, 100 abdominal CT images obtained with a sufficient original dose were adopted for transfer learning. These CT images were obtained from a publicly available dataset (The Cancer Imaging Archive), which is an open-access information resource created by the US National Cancer Institute [15]. Simulated dose-reduction images were generated within these abdominal images, with a 10-step dose reduction rate from the original dose to 1/100. Therefore, the number of training images in the proposed transfer learning was 1000. Subsequently, these images were selected in two cases, and 5% of the data were used to validate the denoising performance during the training. Training images were cropped in 350 × 250 pixels (350 × 500 mm^2^) in the center of the image and randomly divided into small patches of 50 × 50 pixels, with 15 patches per image. Furthermore, the input image was rotated randomly from 0° to 90°, horizontally. These learning methods can prevent overfitting using training datasets. The Adam optimizer was used for training at a learning rate of 10^− 3^, and the networks were trained in 150 epochs. A Quadro RTX 5000 GPU graphics processor (NVIDIA Corporation) was adopted for training and evaluation. The total training time was approximately 10 h.

### CT noise-simulation images

**Fig. 2:**
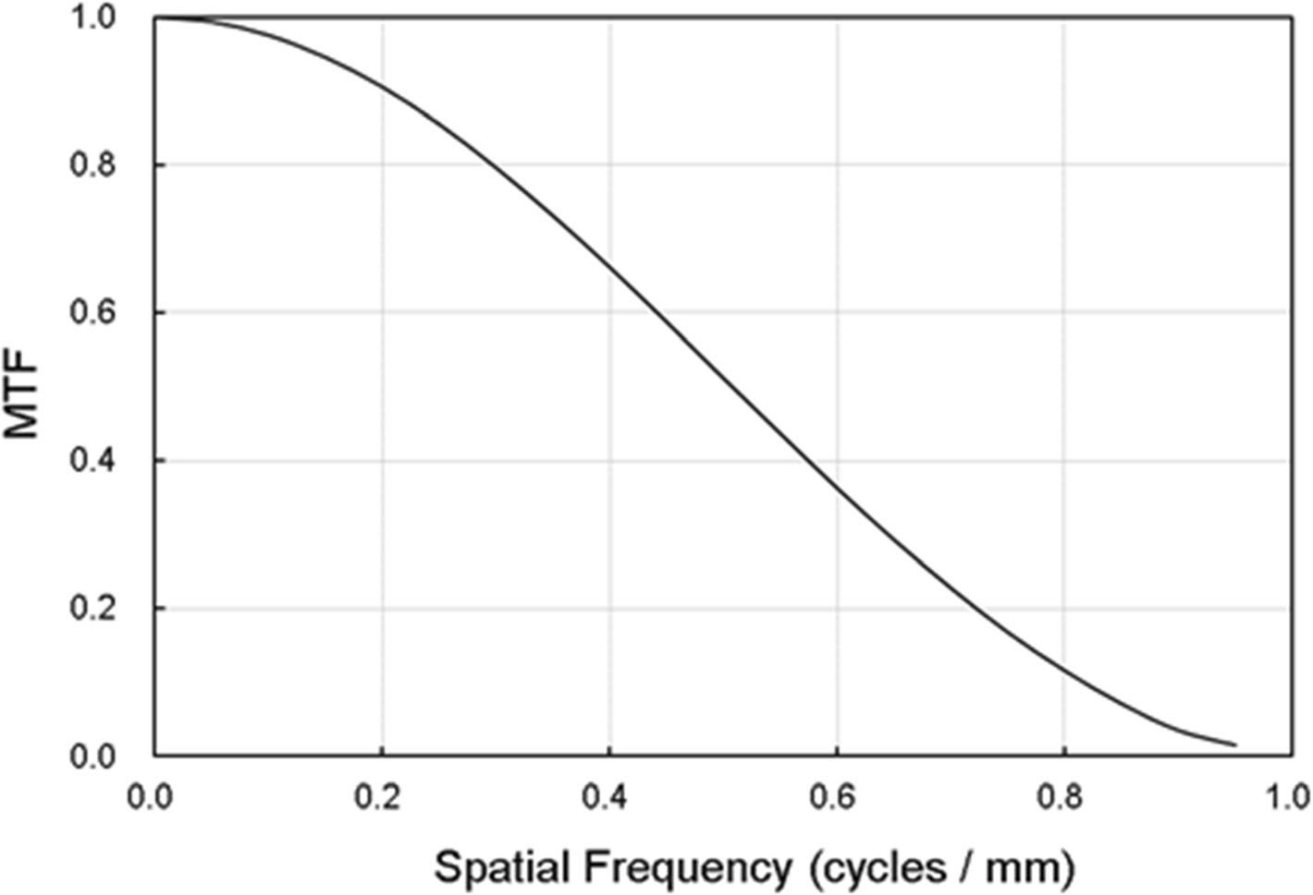
CT unit-specific modulation transfer function

The dominant CT noise statistics in X-ray reduction are known to follow a Poisson distribution. Therefore, to simulate a low-dose CT image, a Poisson noise distribution was introduced while convoluting the CT unit-specific modulation transfer function (MTF) [16]. Figure 2 presents the MTF curve applied during the noise simulation process. By adding these simulated CT specific noises, statistical pixel value deviation images were created according to the incident dose reduction levels. Here, simulated noise were created with standard deviations of 1.3, 2, 10, and 20 times the original image to create 75%, 50%, 10%, and 5% dose-equivalent images. Accordingly, unique reduced-dose images reflecting the CT unit noise characteristics were created for this denoising study.

### Evaluation of image quality

To evaluate the image quality of denoised images using DnCNN, and compare its performance with that of other noise-reduction methods, the CT value intensity was measured, and the structural similarity index (SSIM) and peak signal-to-noise ratio (PSNR) were calculated for the denoised images [17]. The SSIM was calculated as follows:

1 $$ SSIM\left(x,y\right)=\frac{\left(2{\mu}_x{\mu}_y+{C}_1\right)\left(2{\sigma}_{xy}+{C}_2\right)}{\left({\mu^2}_x+{\mu^2}_y+{C}_1\right)\left({\sigma^2}_x+{\sigma^2}_y+{C}_2\right)} $$

where *μ*, *σ*, *C,* and *x*, *y* represent the signal mean, variance, *C* regularization constant, and each evaluation image, respectively. Furthermore, the PSNR was calculated as:

$$ MSE=\frac{1}{M\times N}{\sum}_{i=1}^M{\sum}_{j=1}^N{\left[x\left(i,j\right)-y\left(i,j\right)\right]}^2, $$

2 $$ PSNR=10{\log}_{10}\frac{\mathit{\max}{\left|x\left(i,j\right)\right|}^2}{MSE} $$

PSNR is the maximum value in the input image data [*x* (*i*, *j*)] divided by the mean squared error (MSE) between image *x* (denoised low-dose image) and image *y* (original full-dose image). In addition, *M* and *N* represent the width and height of the images, respectively.

In this study, the performance of DnCNN was compared with other noise-reduction methods using median, Gaussian, and Wiener filters. In the median filter method, the output value is the median value of the 3 × 3-neighboring pixels. In the Gaussian filter method, an isotropic Gaussian smoothing kernel with a standard deviation of 1.0 is applied to a two-dimensional image. In the Wiener filter method, smoothing kernels in the mean and variance of 5 × 5-neighboring pixels were adopted in the denoising process. Moreover, post-transfer-learned DnCNN was compared with these noise-reduction methods, including the original DnCNN. To compare image quality by adopting the indices of PSNR and SSIM, 10 abdominal CT images composed of 350 × 250 pixels (350 × 250 mm^2^) with 3-mm slice thicknesses were used for the quantitative evaluation. These CT images were obtained from a publicly available dataset, from the Cancer Imaging Archive [13]. The simulated noise images were created in 75%, 50%, 10%, and 5% equivalent doses relative to the original dose. The difference between the results of each noise reduction method and the original DnCNN method was considered statistically significant (two-tailed t-test, *p* < 0.05).

## Results

### Denoised images

**Fig. 3:**
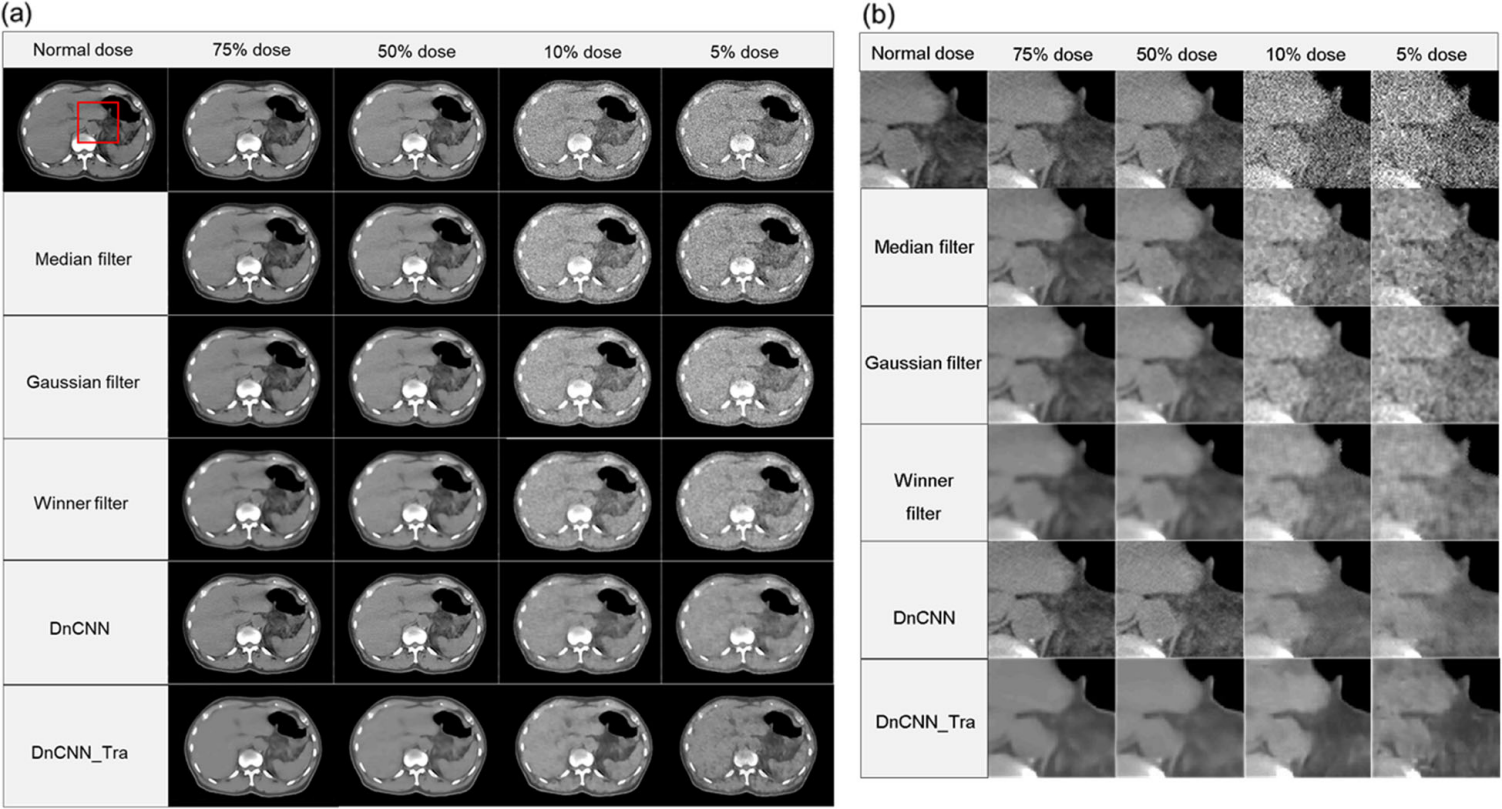
Denoised image with contaminating noise artifacts, which was simulated to minimize dose exposure in the image at four different dose levels. **a** Results of noise reduction in the entire abdominal image; **b** Enlarged images of the region indicated by the red region of interest; all images are presented with the same window width and level. Median, Gaussian, Wiener, original DnCNN, and optimized DnCNN (DnCNN_Tra) techniques were applied for noise reduction at each dose-reduction level

**Fig. 4:**
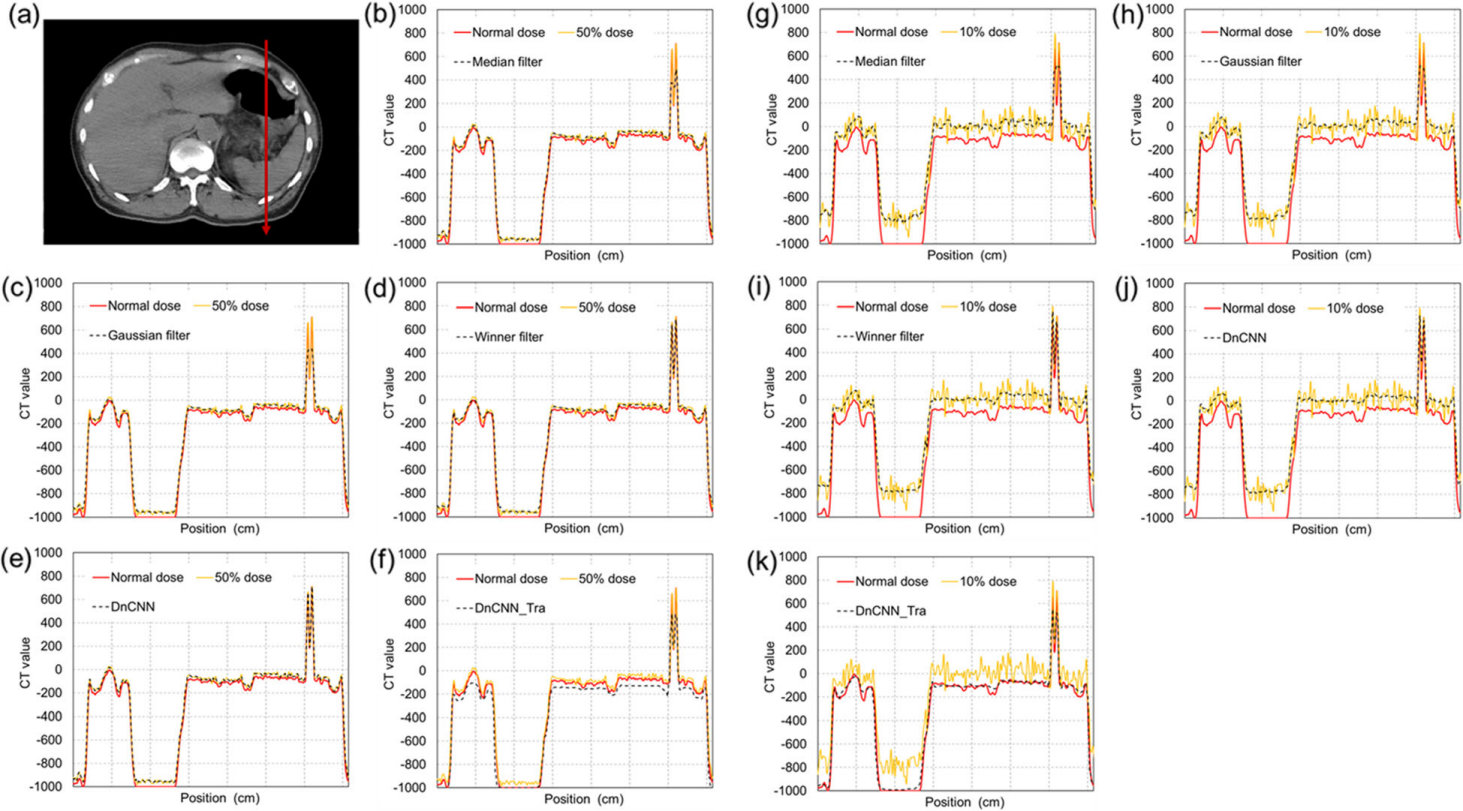
Comparison of the vertical profiles in each image. **a** Profile position; **b-f** CT value profiles along the red arrow in (**a**) with each noise reduction method in the 50% dose equivalent image; **g-k** CT value profiles along the same line in the 10% dose equivalent image. Red line depicts the normal dose image, yellow lines represent the dose reduction images, and each black dashed line denotes the denoised images by the Median, Gaussian, Winner, original DnCNN and DnCNN_Tra techniques, respectively

**Fig. 5:**
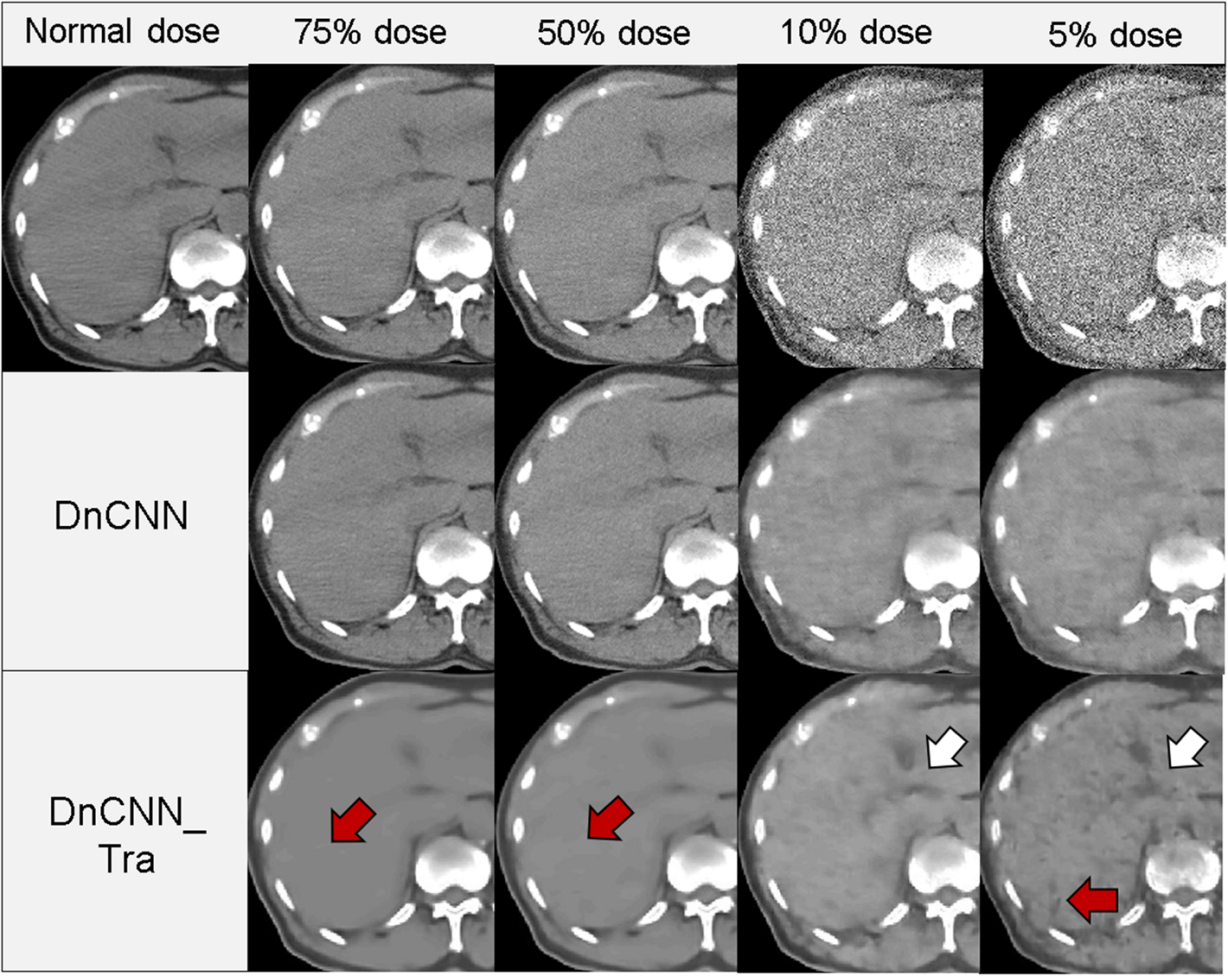
Comparison of the visual inspection by denoising, between the original DnCNN and trained DnCNN methods. White arrows depict the hepatic sickle mesentery, which can be visualized by the denoising effect using the trained DnCNN method. Conversely, red arrows represent excessive smoothing triggered by the denoising

Figure 3 presents one of the results of the denoised images obtained using each filtering and CNN-based method. Figure 3b compares the enlarged images of one region (indicated by the red rectangle) in Fig. 3a. Moreover, a vertical CT value profile of each image with equivalent dose levels of 50% and 10% are plotted in Fig. 4. In these results, DnCNN_Tra represents the application of transfer learning for the original DnCNN model. Regarding the ultra-low-dose levels (equivalent to 10% and 5% doses), significant image degradation was triggered by the simulating the reduction in incident photons. The image quality improvement obtained by the filter-denoising methods at ultra-low doses was insufficient. In contrast, it was also observed that the CNN-based methods effectively reduced the noise at these ultra-low doses. Figure 5 presents a comparison of visual inspection via denoising with the DnCNN method. In the results of the original DnCNN method, although the noise had been eliminated, image blurring was introduced at ultra-low-dose levels. Conversely, denoising using the DnCNN_Tra was adopted to preserve the image edge sharpness, and the hepatic sickle mesentery was visualized, as depicted by the white arrows in Fig. 5. Moreover, the profile of the CT value in the 10% dose equivalent image was similar to that of the normal dose image, as illustrated in Fig. 4k. Nevertheless, excessive smoothing at dose-reduction levels of 75% and 50%, and inaccurate blackout regions at dose-reduction levels of 5% were observed, as indicated by the red arrows in Fig. 5.

### Quantitative evaluation of image quality

**Fig. 6:**
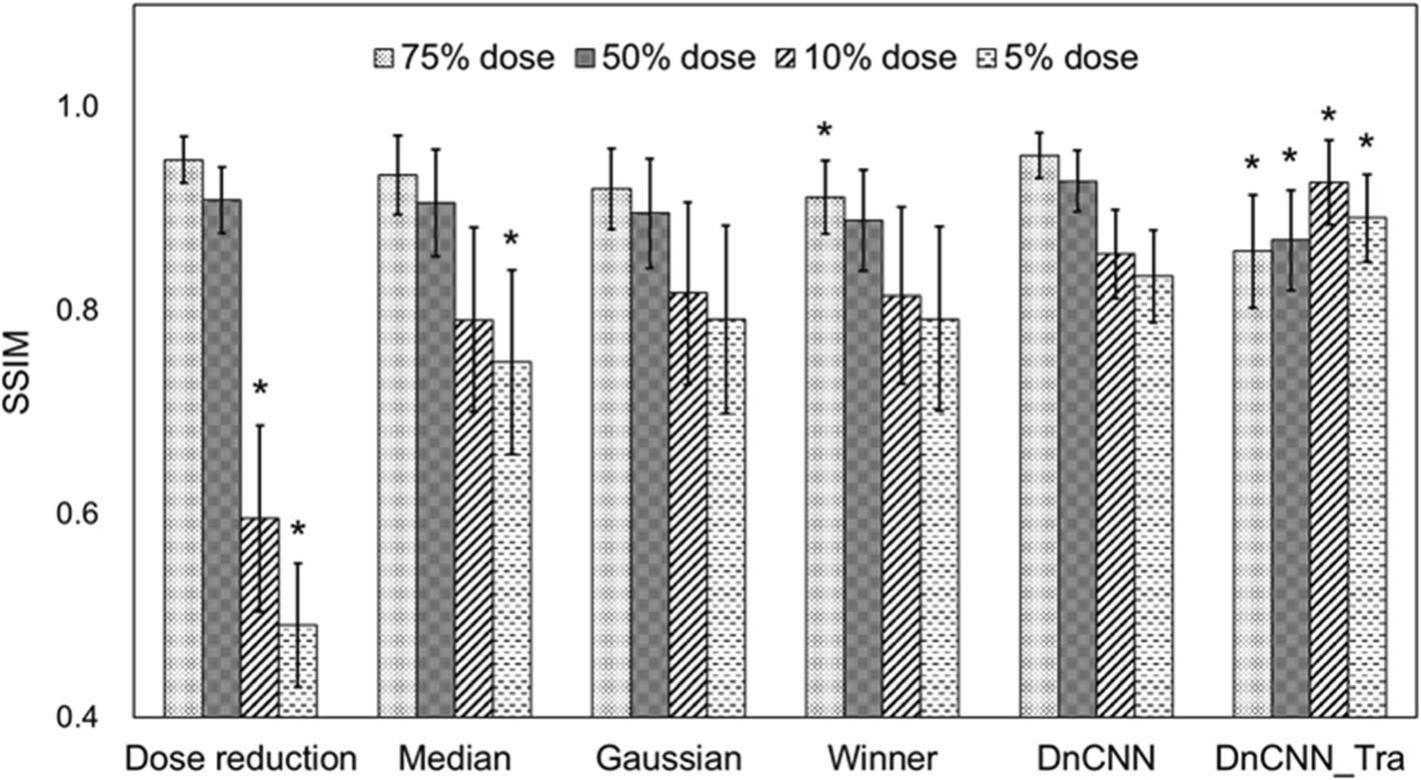
SSIM results for different noise-reduction methods for each dose-reduction simulated image. * indicates *p* < 0.05, which represents a significant difference in the SSIM value relative to the results of the original DnCNN method

In Figs. 5 and 6, the image qualities of the original DnCNN and DnCNN_Tra methods were compared in terms of SSIM and PSNR, respectively, over different dose-reduction levels among noise-suppression filters. For the SSIM values, the DnCNN method tended to provide improvements at all dose reduction levels. However, no significant difference was observed with respect to the results of the Gaussian filter method. In contrast, PSNR results were significantly improved in the 75% and 50% equivalent-dose images when compared with the results of the Gaussian filter method. However, at ultra-low-dose levels, the DnCNN method could not obtain significantly higher PSNR values than the other noise-reduction methods. For DnCNN_Tra, the SSIM was improved by approximately 10% relative to that of the original DnCNN method in the 5% and 10% dose-equivalent images. In contrast, in the results for the 75% and 50% dose-equivalent images, SSIM and PSNR were significantly degraded relative to the results for the original DnCNN method.

## Discussion

In this study, the ability of general pre-trained CNN image denoising to discard noise triggered by the lack of incident photons in CT images, was assessed. For diagnostic and radiation therapy purposes, denoised images should exhibit sufficient quality to facilitate clinically sound decisions. Therefore, the quantitative evaluation of image quality is based on its similarity to normal dose images when comparing noise-reduction methods.

The denoising performance in terms of image SSIM and PSNR was significantly better for DnCNN than for the other image-space denoising methods. In the results presented in Fig. 3, the DnCNN method effectively reduces noise at all dose reduction levels. The results of the image-space filtering methods at ultra-low levels of 10% and 5% dose equivalents were insufficient. These results were triggered by the extremely large deviation in the image pixel values, thus suggesting the limitations of denoising using a reconstructed image space.

As illustrated in Fig. 6, the DnCNN method tended to increase the SSIM value at all dose levels; therefore, an improvement in the overall image similarity is expected. Although the DnCNN model was pretrained on 400 grayscale images and a Gaussian noise level of 25 for natural image denoising [10], its potential effectiveness for denoising low-dose CT was also validated in our study. However, there were no significant differences in the SSIM values between the results of the DnCNN and Gaussian methods. A virtual dose-reduced image was simulated according to Poisson distribution. Accordingly, the Gaussian filter method was effective in removing noise in this study. In contrast, because the DnCNN model also learned a Gaussian distribution, effective results were obtained when removing noise statistics from low-dose CT images.

**Fig. 7:**
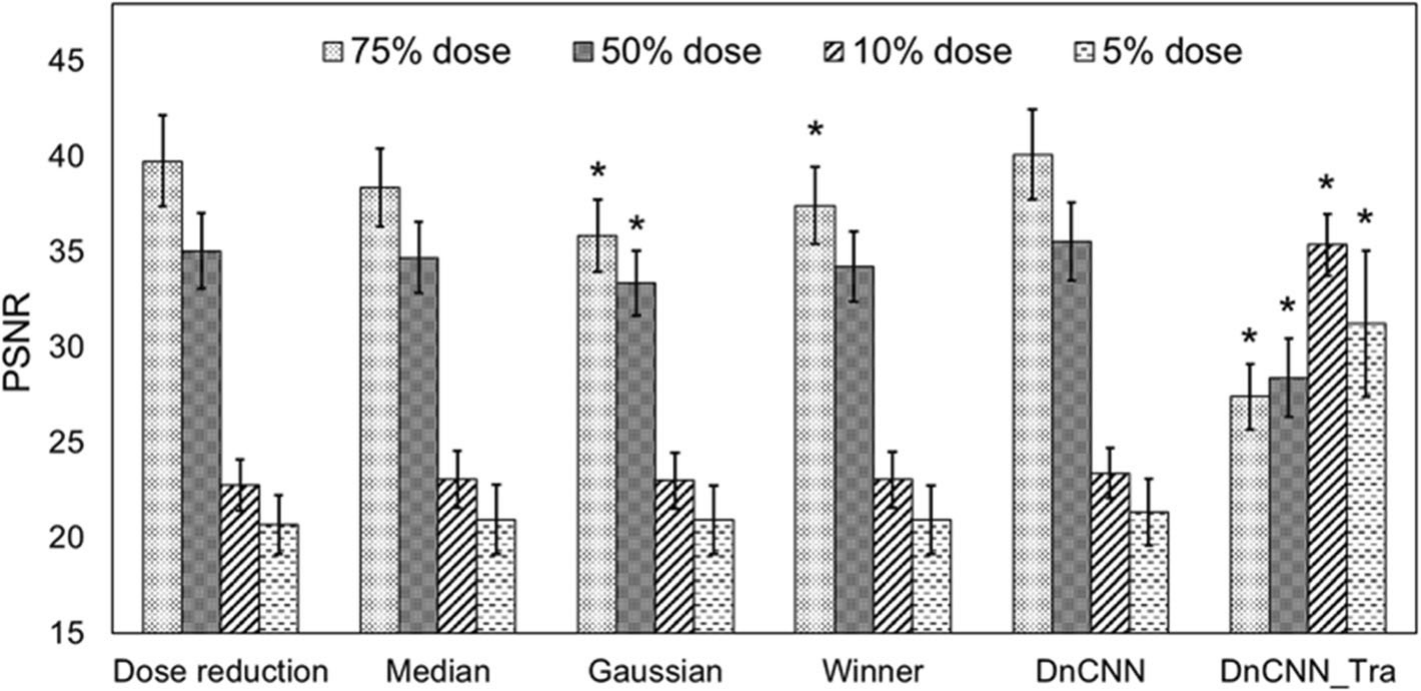
PSNR results for different noise-reduction methods of each dose-reduction simulated image. * indicates *p* < 0.05, which represents a significant difference in PSNR value relative to the results of the original DnCNN method

The results in Fig. 7 indicate that the DnCNN method realizes significantly better results than those of the Gaussian model at the 75% and 50% dose-equivalent levels. However, at ultra-low-dose levels, no significant improvement was observed relative to the other noise-reduction methods. The PSNR value is calculated as the noise component divided by the MSE of the pixel values of the normal-dose and denoised images. Therefore, both insufficient denoising and excessive smoothing trigger deviations in pixel values from the original image. In this case, PSNR is degraded. PSNR values obtained by the DnCNN method were influenced by the excessive smoothing, and these results can be visually observed in Fig. 6. In contrast, because a significant improvement compared with the results of the Gaussian method can be demonstrated, the DnCNN method eliminates noise more naturally in CT image denoising than other methods. However, it has been observed that this over-smoothing behavior is associated with the original DnCNN in denoising very low-dose CT data. This is the same tendency as described in a previous study [8]. The CT value of the internal body was overestimated by applying the original DnCNN method (Fig. 4j). Conversely, the DnCNN_Tra was able to eliminate noise and maintain image sharpness at the 10% and 5% dose-equivalent levels; therefore, the clinical anatomical structure (hepatic sickle mesentery) could be visualized (white arrows in Fig. 5). Moreover, the CT profile agreed well with that of the normal-dose image (Fig. 4k). Therefore, SSIM and PSNR results were significantly increased relative to those of the original DnCNN. Therefore, the current denoising model, which was pre-trained on natural/synthetic images using Gaussian noise, can be updated by simulating dose-reduction images. However, at dose-equivalent levels of 75% and 50%, the results of the DnCNN_Tra images also exhibited excessive smoothing (red arrows in Fig. 5). This excessive smoothing triggered pixel value deviations from the original dose image; therefore, SSIM and PSNR values decreased in 75% and 50% dose-equivalent images compared to those in 10% and 5% images. Moreover, several inaccurate blackout regions occurred in the results for the 5% dose-equivalent image (red arrows in Fig. 5). Accordingly, low-dose CT with dose-reduction levels of ≤ 50% is sufficient when using the original DnCNN method, and 50% to 10% dose-equivalent images are adequate for adopting the trained DnCNN model for image denoising. Moreover, CNN-based denoising of images at dose equivalent levels of < 10% has a high risk of generating new image artifacts. Therefore, CNN-based denoising for images with dose-equivalent levels of < 10% from the original dose cannot create accurate noise reduction images. The proposed transfer learning could not have performed well at all dose reduction levels. The distribution of high-noise components is possibly significant for the training data. Moreover, residual learning of Poisson distribution-based CT-specific noise features may have resulted in excessive smoothing. In the transfer learning process, the pre-trained learning model with prior data was maintained, and only the last fully connected layer was updated for the new data. Therefore, the CT noise became excessive, thus resulting in over-smoothing, which occurred in the high-dose region with less noise areas. The deeper the layer, the more abstract the learned feature. This can be attributed to the lack of new networks for extracting important features from training images, which is a result of the small amount of noise variation data used in training them [18, 19]. In previous studies, the reconstruction of the learning mechanism for a specific purpose was achieved by optimizing the learning model by transfer learning [20–22]. In these studies, training data were experimentally created using human image and phantom data; therefore, the accuracy of their noise features was realistic and of high quality. Moreover, adjustments in the noise distribution of the training data and improvements in the CNN architecture are required to reduce noise at any dose level in clinical CT images.

The limitations of this study include the need to train and evaluate the denoising accuracy of the DnCNN method in actual low-dose exposure images. However, it is impossible to obtain CT images with the same organ positions without any misalignment obtained at various dose levels for model learning. In this study, the noise-removal capability of the DnCNN method can be quantitatively demonstrated using unique CT noise-simulation images. In contrast, the accuracy of this CT noise simulation will influence the results of CNN-based denoising, and realistic CT noises will increase the accuracy of the deep learning model.

## Conclusions

The proposed DnCNN-based denoising method adequately denoised the CT-specific noise resulting from low-dose X-ray exposure. Furthermore, the denoising properties of the proposed method are more suitable than those of other noise-reduction methods, particularly at ultra-low-dose levels. Transfer learning with tailored DnCNN facilitated the elimination of image noise, and prevented over-smoothing at ultra-low doses. In addition, it improved image similarity by approximately 10%. However, dose-equivalent levels of < 10% of the original dose could not create accurate noise reduction images. Developing a generally applicable denoising network via optimal network design and training data modification is required for appropriate noise reduction at all noise levels.

## Data Availability

The datasets generated and analyzed during the current study are available in the Cancer Imaging Archive repository (https://www.cancerimagingarchive.net/).

## Abbreviations

CT: Computed tomography
CNN: Convolutional neural network
Dn: Denoising network
SSIM: Structural similarity index
PSNR: Peak signal-to-noise ratio
ReLU: Rectified linear units
MTF: Modulation transfer function
MSE: Mean squared error
BN: Batch normalization
